# Imaging of the Spinal Cord in Multiple Sclerosis: Past, Present, Future

**DOI:** 10.3390/brainsci10110857

**Published:** 2020-11-13

**Authors:** Yongsheng Chen, Ewart Mark Haacke, Evanthia Bernitsas

**Affiliations:** 1 Department of Neurology, Wayne State University School of Medicine, Detroit, MI 48021, USA; ys.chen@wayne.edu (Y.C.); ehaacke@med.wayne.edu (E.M.H.); 2 Department of Radiology, Wayne State University School of Medicine, Detroit, MI 48021, USA

**Keywords:** multiple sclerosis, spinal cord, magnetic resonance imaging, spinal cord atrophy, magnetization transfer ratio, diffusion imaging, disability

## Abstract

Spinal cord imaging in multiple sclerosis (MS) plays a significant role in diagnosing and tracking disease progression. The spinal cord is one of four key areas of the central nervous system where documenting the dissemination in space in the McDonald criteria for diagnosing MS. Spinal cord lesion load and the severity of cord atrophy are believed to be more relevant to disability than white matter lesions in the brain in different phenotypes of MS. Axonal loss contributes to spinal cord atrophy in MS and its degree correlates with disease severity and prognosis. Therefore, measures of axonal loss are often reliable biomarkers for monitoring disease progression. With recent technical advances, more and more qualitative and quantitative MRI techniques have been investigated in an attempt to provide objective and reliable diagnostic and monitoring biomarkers in MS. In this article, we discuss the role of spinal cord imaging in the diagnosis and prognosis of MS and, additionally, we review various techniques that may improve our understanding of the disease.

## 1. Introduction

Multiple sclerosis (MS) is a chronic, inflammatory, autoimmune, neurodegenerative disease characterized by a wide range of symptoms. Pathological damage in the central nervous system (CNS), from the brain to the spinal cord (SC), is common in different stages of MS [1,2]. As part of the CNS, the SC connects neurons in the brain down to peripheral nerves via ventral (primarily motor nerve) and dorsal (primarily sensory nerve) nerve roots. The tubular-like SC is surrounded by cerebrospinal fluid (CSF) through all segments from C1 to approximately the L1/L2 vertebral level. In imaging, the transverse section of the SC shows symmetrical peripheral white matter fibers with a central butterfly or H-shaped grey matter (GM) region [3]. 

The cross-sectional area (CSA) of the SC typically ranges from 67 to 101 mm^2^ at the cervical level in adulthood [4,5,6]. These dimensions and the anatomical configuration of the SC make magnetic resonance imaging (MRI) one of the most suitable techniques to image the SC in vivo and it has been widely used for decades in clinical and research settings. MRI provides rich image contrast which comes from the differences between the MR tissue properties such as proton density (PD), longitudinal relaxation time (T1), transverse relaxation time (T2), and the molecular environment. Typical SC lesions in MS appear most commonly in the cervical area and mainly in the periphery of the SC, as focal or diffuse hyperintense lesions on T2 weighted (T2W) images caused by increased water content and prolonged T2 during the inflammatory and demyelination process. 

A number of studies have suggested that SC lesions and atrophy are stronger predictors of disability in MS than other measures, however, no consensus has been reached on which methods provide the best measures. [1,2,7,8,9,10]. Despite the fact that various qualitative and quantitative MRI metrics have been used over the past two decades in both routine practice and research in diagnosing MS and its progression, there are still several major challenges related to sensitivity, specificity, reproducibility and reliability. These technical challenges include: image artifacts caused by respiratory and cardiac motion; swallowing and CSF pulsations; fatty tissue influence; susceptibility artifacts at air/tissue interfaces when a gradient echo-based sequence is used; mobility of the SC; insufficient signal-to-noise ratio (SNR); imaging resolution; and the lack of normative data. 

In this article, we discuss the clinical relevance of SC MRI in the diagnosis, prognosis and response to treatment in MS. Moreover, we review various MRI techniques used for imaging the SC and its involvement in MS. Finally, we present the most recent advances and the use of ultra-high field SC MRI in MS. 

We initially searched PubMed from the year 2000 onward using the following terms for papers written in the English language: (‘MS’ or ‘multiple sclerosis’) and (‘MRI’ or ‘MR’ or ‘magnetic resonance’) in the ‘title’ as well as ‘spinal cord’ by itself being in the ‘title’ or ‘abstract’. The search resulted in 540 articles, including 126 review articles. Since we were aiming to prepare a narrative review in the current topic, we reviewed the abstracts of most of these articles, and selected relevant citations for each sub-topic based on our knowledge. In addition, we identified more articles from the references of the articles we reviewed.

## 2. Spinal Cord MRI and the McDonald Criteria in MS Diagnosis

The SC is one of four areas of the CNS required to document dissemination in space (DIS) and dissemination in time (DIT) given in the McDonald criteria for diagnosing MS [11]. The 2017 McDonald criteria included both symptomatic and asymptomatic SC lesions, while only asymptomatic lesions appear to account for DIS and DIT in previous versions (2001, 2005 and 2010) [12]. As part of the diagnostic criteria, DIS can be demonstrated by one or more T2-hyperintense lesions seen on MRI in at least two of the four regions of the CNS: the SC, periventricular, juxtacortical and infratentorial brain regions, while DIT is associated with new or gadolinium-enhancing lesions [11]. Although brain MRI imaging is most commonly used in MS diagnosis and/or tracking the disease progression, SC imaging is recommended for a complete study (at least at the cervical level) whenever possible [1,2,11,13,14]. Further, SC MRI is also recommended in MS patients with a primary progressive (PPMS) course, in cases where MS is not commonly diagnosed (older patients) and where the presentation is non-specific and the diagnosis needs to be further supported. 

SC MRI is more straightforward than brain MRI in fulfilling the McDonald criteria and differentiating MS from MS mimics. A recent study demonstrated that the MRI DIS criteria in the brain are often misunderstood and misapplied both by neurology residents and MS specialists. T2 hyper-intense lesions in the brain were incorrectly identified by 61% of residents and 48% of specialists for periventricular lesions, and incorrectly identified by 72% of residents and 47% of specialists in juxtacortical lesions; in contrast, a majority of these participants correctly assessed SC MRI [15]. Moreover, T2 hyper-intense lesions in the brain are non-specific to MS, while SC lesions are more specific and contribute to a more accurate diagnosis of MS. Therefore, there is strong motivation to continue to refine and apply SC MRI in MS studies. 

## 3. Spinal Cord Atrophy

A number of pathological processes, such as axonal loss, demyelination, and gliosis contribute to spinal cord atrophy in MS [16]. Spinal cord atrophy can be seen in early MS, even in cases of clinically isolated syndrome (CIS); however, results are conflicting [9,17]. Traditionally, the measures of cervical cord CSA along with correlations to disability measured by the expanded disability status scale (EDSS) are typically used in MS studies to reflect SC atrophy [4,5]. Given the limitation of imaging resolution and the resulting partial volume effects, physiological variation, and the different sequences used in measuring CSA of the cervical cord in different studies, a consensus is yet to be reached on how to use SC CSA as a primary outcome measure in clinical trials. Two recent systematic reviews and meta-analysis articles concluded that SC atrophy measured by cervical cord CSA moderately correlated with clinically measured disability in MS [6,9]. The early work by Losseff and colleagues demonstrated reproducible CSA measurements at the cervical cord level using an inversion recovery (IR)-based T1 weighted (T1W) sequence, and described a strong correlation (*r* = −0.7, *p* < 0.001) between cervical cord CSA and EDSS in a medium sized cohort (*n* = 60) of MS patients [4]. In another study involving 150 patients with relapsing-remitting MS (RRMS) and secondary progressive MS (SPMS) phenotypes, a strong correlation (*r* = −0.75, *p* < 0.001) was found between cervical cord CSA and EDSS in all MS patients, and a sub-group analysis showed significant correlations both in RRMS (*r* = −0.38, *p* = 0.004, *n* = 93) and SPMS (*r* = −0.4, *p* = 0.0021, *n* = 57) along with a significant difference in CSA mean values between the two sub-groups, In this study, CSA was an independent predictor of disability in both MS phenotypes [5]. Longitudinal studies demonstrated an overall 1.78% annual decrease in cervical cord CSA in patients with MS, and 2.08% in progressive MS, a much higher rate than the annual brain volume loss in MS, which is estimated at between 0.5% and 1% [6]. However, other studies reported very weak correlations (*r* > −0.3) between cervical cord CSA and EDSS in MS [18,19,20,21,22]. 

Despite the large variation of correlations between SC CSA and disability scores in the literature, cervical cord CSA robustly measures SC atrophy in MS versus controls, with notable progression in progressive phenotypes, thereby providing a potential viable objective measure relevant to clinical outcomes. In addition, it is quite common in a brain imaging session to cover the cervical cord down to the C2/C3 level, making cervical cord CSA measurements clinically practical with automatic segmentation tools. Finally, a recent 3T study using combined imaging of the brain and SC has reported the cervical cord GM T2 lesion load and the fractional anisotropy value in the lateral funiculi as independent predictors of disability in RRMS and the cervical cord GM CSA as an independent predictor of disability in progressive MS [23]. 

Over the last decade, overcoming traditional limitations of CSA measurement has become a major focus in SC imaging. A new technique, the generalized boundary shift integral (GBSI), is a registration-based measurement of spinal cord atrophy that has overcome past challenges and measures cord atrophy more accurately than segmentation-based techniques. In a comparative longitudinal study, GBSI showed less variability than CSA, similar rates of cord atrophy and better differentiation between healthy controls and different subtypes of MS patients. The results of these studies support the use of GBSI, especially for longitudinal studies, as higher accuracy in SC atrophy measurement can be achieved [24,25]. In a similar study in PPMS patients, the GBSI-based measurements of spinal cord atrophy were clinically meaningful in a relatively low sample size, making GBSI cord atrophy a potential secondary outcome measure in clinical trials in MS [26].

## 4. Spinal Cord MRI in Multiple Sclerosis

It has been over three decades since the initial application of SC MRI in MS [27]. To date, qualitative assessment of SC lesions has been utilized in the current consensus of MS diagnostic criteria for demonstrating DIS and DIT [11,13,14]. However, today, quantitative MRI techniques are used more and more in assessing disease progression and determining prognosis [28]. In addition, spinal cord MR spectroscopy (MRS) and functional MRI (fMRI) have started to be used in the research setting [29]. 

### 4.1. Qualitative MRI

#### 4.1.1. Standardized SC MRI Protocol in MS

In the standardized SC MRI protocol, a dual-echo turbo spin echo (TSE) sequence is commonly used to provide a proton density weighted (PDW) image from the short echo time, along with the standard T2W from the second echo. A PDW image is expected to better depict diffuse lesions in the cord than the T2W image [30]. Given that the T2 weighted fluid attenuated inversion recovery (FLAIR) image is not sensitive in detecting white matter SC lesions [31,32,33], either a short-tau inversion recovery (STIR) image or T1 weighted IR image are used [13,14]. In the latter case, an inversion time (TI) is chosen to null the signal of normal-appearing SC to increase lesion contrast thanks to the difference in their T1 values [34,35,36,37,38]. To null the normal-appearing cord tissue, the inversion time is chosen to be roughly 69% of the normal-appearing SC T1 value. Poonawalla et al. demonstrated that 40% of SC lesions clearly detected by this optimized T1 weighted IR sequence were not visible on the PDW and T2W images from the dual-echo FSE scan [34]. Another means to enhance contrast is using phase-sensitive inversion recovery (PSIR). Compared to the STIR, PDW and T2W image, the PSIR images have been shown to have significantly higher lesion to normal-appearing spinal cord contrast [34]. Furthermore, the PSIR sequence was superior in visualizing diffuse lesions than STIR [34,39]. The PSIR reconstruction takes advantage of the slower growth of long T1 components and uses the sign (whether the tissue is positive or negative) to increase the contrast between lesions and normal-appearing spinal cord. It avoids the loss of contrast for subtle diffuse lesions, suppresses the signal from CSF, and creates hypo-intense lesion intensity. Therefore, the PSIR image is recommended along with the conventional magnitude reconstruction. 

A group of clinicians and scientists representative of the American Academy of Neurology, the Radiological Society of North American, and the American Society of Neuroradiology have summarized a standardized SC MRI protocol which is recommended if the brain MRI is not sufficient to demonstrate DIS or if the symptoms are related to the SC [13]. This SC MRI protocol consists of a T1W, PDW, and either a STIR or PSIR set of sequences collected in the sagittal plane, and a T2W scan collected in the axial plane focusing on the lesion segments [13]. Gadolinium-enhanced T1W is also recommended in this protocol. The European collaborative research network also has a consensus, MRI in multiple sclerosis (MAGNIMS), for appropriate criteria in diagnosing MS [14]. The MAGNIMS protocol recommends SC MRI to demonstrate DIS if patients have clinical isolated syndrome (CIS) suggestive of SC involvement. The protocol proposes using T2W, STIR, double inversion recovery (DIR), and gadolinium-enhanced T1W for imaging the entire SC since a notable portion of SC lesions in MS are associated with the thoracolumbar segments [14,40,41,42].

#### 4.1.2. Emerging Sequences in Addition to the Standardized SC MRI Protocol

The present standard T1 weighted sequence in the brain imaging, coined T1 weighted magnetization prepared rapid gradient echo (T1W-MPRAGE), which is an IR-based 3D sequence, has also been optimized to image SC lesions in MS [37,42]. A study by Nair et al. demonstrated that T1W-MPRAGE was able to depict focal and diffuse lesions in the cervical and thoracic SC more conspicuously (with better contrast-to-noise ratio (CNR)) than other contrasts like STIR, T2W and T2*W [42]. A second IR-based sequence sensitive to intracortical MS lesions in the brain, DIR, has been suggested for imaging MS SC in a study by Riederer and colleagues in a clinical 3T setting [43]. It also has superb lesion detectability compared to conventional T2W [43]. The beauty of this approach is that both normal appearing SC and CSF are suppressed, leaving a hyper-intense lesion, while the major difficulty is the low SNR in the image. However, it is not widely used in clinical practice because it is easily affected by artifacts and magnetic field inhomogeneities and takes a long time to acquire the data. MP2RAGE is another IR-based sequence with two gradient echo acquisition kernels following the inversion pulse, that has been used in the brain to quantify T1 [44] and has recently been used in imaging SC lesions in MS [45]. The latter study showed improved visibility of cervical lesions compared to STIR and T2W, with a 62% increased detection of lesions at 3T in a cohort of patients with all MS phenotypes, including RRMS, SPMS and PPMS [45]. 

### 4.2. Quantitative MRI

Quantitative MRI techniques have been studied in imaging the SC in MS including the quantification of relaxometry, proton density, magnetization transfer, and microscopic water proton motion via diffusion, to name a few of interest to imaging the SC in MS (Table 1). These quantitative MRI approaches provide great potential in monitoring disease progression when compared to structural measures such as CSA, cord volume, or lesion load. 

#### 4.2.1. Relaxometry and Proton Density

The quantification of relaxometry including T1, T2, and T2*, as well as PD, are well established in the field of brain MRI, but have been less investigated in imaging the SC. These quantitative metrics provide not only objective assessment of pathological lesions across subjects, instruments and time points but also the basis for other quantitative approaches. Moreover, the optimization of imaging parameters to obtain the best image contrast needs the precise knowledge of relaxivities in normal-appearing tissues and pathologies. By varying one imaging parameter such as inversion time, repetition time (TR), echo time (TE), or flip angle, a set of data acquired with all the other imaging parameters unchanged can be used for fitting tissue properties for T1, T2, T2* and PD, respectively [57]. 

Rasoanandrianina and colleagues optimized the MP2RAGE sequence for in vivo SC T1 mapping at 3T [58]. This method uses a look-up table to estimate T1 values relative to the MP2RAGE signal intensity which is insensitive to radiofrequency fields and has been well-established in mapping the brain [58,59]. The T1 values of dorsal and lateral columns measured by MP2RAGE were 925 and 888 ms, respectively [58]. Despite the discrepancy of regional T1 values of the SC from these studies [58,59,60,61] which only involved a few healthy control subjects, clinical investigations of quantitative T1 assessment in MS population are warranted. Up to the time of submission of this manuscript, there are no studies on MS using relaxometry and PD for SC imaging.

#### 4.2.2. Magnetization Transfer

Magnetization transfer (MT) MRI utilizes the frequency difference between bound water protons in macromolecules and free water protons and their interactions. Generally, an MT pulse is applied to selectively saturate the bound pool, thereby modifying the contrast between tissues with different macromolecular content. Myelin-rich tissue such as white matter (WM) has restricted water motion and a broad range of resonance frequency offsets relative to the resonance frequency of free water protons. By using an off-resonance radiofrequency pulse to prepare the magnetization, the consequent imaging kernel produces saturated signals for tissues with abundant macromolecules, giving images with magnetization transfer contrast (MTC). Consequently, demyelinated WM tissues are less saturated than normal myelinated WM tissues. This myelin sensitive MT imaging is more specific to demyelination in spinal cord lesions than qualitative contrasts which reflect not only demyelination but also inflammation, increased water content, edema, gliosis and axonal loss.

The first large MS cohort study using SC MTR by Filippi and colleagues observed overall significantly lower MTR of the cervical cord in MS than healthy subjects. Rather than looking into lesions and normal-appearing cord MTR values, the study used a histogram-based analysis on the entire cervical cord. There were no significant differences of histogram-based MTR measures between patients with RRMS and healthy controls, but patients with PPMS had significantly lower mean MTR and peak height than controls. Patients with SPMS also had significantly lower peak height than patients with RRMS, suggesting that MTR could be an independent predictor of motor disability [46]. Oh et al. aimed to correlate MTR of the SC with clinical disability levels in patients with MS mimicking the clinicoradiologic paradox. Their study categorized a large cohort of MS patients into four subgroups by low- and high-lesion loads counted on conventional MRI combined with low- and high-disability levels assessed by EDSS scores. They found that the MTR values measured over the whole cord cross-sectional images at the C3−4 level were significantly lower in the low-lesion high-disability subgroup than those in low-lesion low-disability subjects, while there was no significant difference in the two subgroups with high-lesion loads [50]. An explanation is that MTR reflects the overall myelin density and, thereby the degree of demyelination. When there is high lesion load in the SC, the axonal loss dominates the level of disability and the myelin-sensitive measures are then less strongly correlated with EDSS scores. The main problem with a significant number of MTR methods is that although the means between two matched populations can be significantly different, there is often a major overlap of the two distributions; therefore, the use of this particular method for MS prognosis on an individual basis is quite limited. 

Calculating MTR requires two scans with and without an MT pulse and has a lower SNR than the original images. Furthermore, it requires image co-registration due to the mobility of the SC. Smith and colleagues proposed using CSF-normalized MT (MTCSF) to quantitatively assess the MT effect in the SC since CSF is insensitive to MT pulse and surrounding the cord on all image slices [62]. In a well-designed study, Zackowski and colleagues utilized the MTCSF for column-specific analyses in MS patients to distinguish abnormalities in individual WM columns of the cord related to specific functions. Compared with controls, the column-specific analyses showed that the SPMS cohort had significantly higher lateral and dorsal columns MTCSF as well as grey matter of the cord. Only the lateral column in the PPMS cohort had significantly higher MTCSF values than controls, while none of the column-specific MTCSF values reached statistical differences in the RRMS cohort. Sensorimotor abnormalities in MS patients moderately correlated with MTCSF signal intensities of the relevant column suggesting that the MTCSF signals have increased specificity for demyelination. In all disease subtypes, both lateral column and posterior column MTCSF were significantly correlated with EDSS. The study introduced a new biomarker, the column-specific MTCSF, that not only correlates with sensorimotor disability but also explains their variance [48]. In a recent longitudinal study using a wide spectrum of quantitative SC MRI measures by Oh and colleagues, to assess disease progression over 5 years in RRMS and progressive MS, SC MTR at C3-4 level was the only measure significantly decreased at 2 and 5 years, followed by CSA that was significantly decreased only at 5 years MS [55]. The subject-specific longitudinal changes in cervical cord MTR correlated with disability measured by follow-up EDSS scores. The study also reported strong correlations between the 2 year and 5 year subject-specific MTR changing rates suggesting that the 2-year course changes could be used to predict the long-term progression in MS [55].

#### 4.2.3. Diffusion Imaging

Diffusion weighted imaging (DWI) and diffusion tensor imaging (DTI) provide morphological, pathophysiological, and functional information about biological tissues. DWI utilizes strong directional gradients to diphase molecular water motion (diffusion), thus it reflects not only the water content but also the integrity of the biological tissue’s microstructure [63]. Similarly, DTI uses diffusion gradients in many (at least six) directions to acquire a diffusion tensor matrix that can be mathematically calculated as three orthogonal eigenvectors ($\lambda_{1},\;\lambda_{2},\;\lambda_{3}$) reflecting the dominant water motion direction ($\lambda_{1}$) in biological tissue and the minor motion in the two perpendicular directions ($\lambda_{2},\;\lambda_{3}$). The major eigenvector ($\lambda_{1}$) is typically used as axial diffusivity (AD), while the average of the other two eigenvectors ($\lambda_{2},\;\lambda_{3}$) is called radial diffusivity (RD), and the mean value of the three eigenvectors is the mean diffusivity (MD). The fractional anisotropy (FA) ranging from 0 to 1 calculated with these three eigenvectors is used to reflect the degree of fiber organization. Diffusion kurtosis imaging (DKI), a non-zero statistical index, can be calculated from a set of data with different b-values reflecting the heterogeneous diffusion environments and the degree of diffusion restriction of the tissue [64]. To emphasize slow diffusion in neuronal tissues, a set of high b-values are used with q-space analysis to calculate the displacement distribution function of water molecules for a given diffusion time, so-called q-space imaging (QSI) [65]. Multi-compartment modeling of diffusion MRI, neurite orientation dispersion and density imaging (NODDI) and spherical mean technique (SMT), two other DTI analysis methods, are also of interest in imaging the SC [66,67,68]. These various diffusion imaging metrics have been broadly used in assessing the integrity and microstructure of the SC in MS [47,50,51,53,55,56,69,70,71,72,73,74,75,76,77,78,79]. Over the years, a number of different diffusion imaging models of the spinal cord have been used. 

DTI: Valsasina and colleagues assessed DTI metrics on a 1.5T MRI in a large cohort of MS patients. The study demonstrated that FA and MD histograms of the SC were significantly different in MS compared to controls, and correlated with clinical disability measures [47]. Oh et al. employed DTI on a 3T MRI to assess disease progression in RRMS and progressive MS, but there were no significant changes captured at 2 and 5 years for all DTI metrics [55]. However, the subject-specific longitudinal changes on DTI indices were correlated with disability measured by EDSS. 

QSI: Cortese and colleagues studied the longitudinal changes of QSI metrics as markers of neurodegeneration and independent predictors of disability progression in patients with early PPMS [56]. They found that higher perpendicular diffusivity derived from QSI at baseline was associated with clinically measured disability at 3 year follow-up. The QSI changes in patients with PPMS were independent of the decrease in cord CSA and the number of new cord lesions. Similarly, another study using QSI also found that increased perpendicular diffusivity in the columns and the whole cord was associated with increased disability scores [79]. These studies suggest that QSI has the potential to serve as a monitoring biomarker in MS which reflects microstructural abnormalities in the SC.

DKI: In one of the few studies that investigated global and regional GM and WM microstructural abnormalities in RRMS using DKI and DTI, Raz and colleagues reported significantly elevated global MD and significantly decreased global FA and mean kurtosis (MK) in RRMS, compared with controls. All three parameters differentiated lesions from normal-appearing spinal cord tissue. Notably, FA, MD and MK were able to differentiate patients with low and high EDSS scores using global and GM regional analyses, but none of them worked for WM regional analysis [51]. These findings suggest that DKI is more specific for assessing GM microstructural abnormalities than MD and FA and can provide additional and complementary information to DTI. 

NODDI: Unlike other multi-compartmental diffusion models, NODDI only uses two b-values for mapping neurite orientation, density and dispersion [80]. Collorone and colleagues evaluated the clinical relevance of spinal cord NODDI indices at 3T and found that the neurite density index (NDI) of WM was significantly lower in patients with RRMS than those in controls, suggesting dominant axonal degeneration. Interestingly, the lower NDI values in the spinal cord, but not in the brain, correlated significantly with greater disability measured by EDSS [53]. In a comparative study, By and colleagues used NODDI indices with DKI and DTI in a small cohort of RRMS and demonstrated the reproducibility of these diffusion metrics. Although DKI-derived mean and radial kurtosis, DTI-derived FA and RD were all significantly different between RRMS and controls, the NODDI-derived orientation dispersion index demonstrated superb contrast between normal-appearing WM and lesions in MS [81]. The same group demonstrated the feasibility and reproducibility of SMT in SC imaging and observed decreased apparent axonal volume fraction in normal-appearing WM and lesions in a small cohort of RRMS patients [68]. These various diffusion models provide meaningful quantitation reflecting underlying microstructural changes in the SC that are potential viable diagnostic or monitoring biomarkers in MS.

#### 4.2.4. Myelin and Myelin Water Imaging

In the CNS, oligodendrocytes in the CNS form a myelin sheath surrounding the axons. Direct imaging of myelin, a major portion of CNS, or the ratio of myelin protons relative to the total tissue protons could be very specific in demyelinating disorders such as MS. Pathological changes including demyelination, axonal degeneration, and disruption of myelin junctions could alter the myelin bilayer trapped water content and the intra- and extra-cellular water content in biological tissue. A few emerging MRI techniques are more specific to myelin-related pathologies in MS, including myelin water fraction (MWF), ultrashort echo time (UTE) sequences, and quantitative susceptibility mapping (QSM), to name a few. MWF employs multiexponential fitting on multi-echo data to extract the short T2 compartment along with the long T2 compartment of the signal [82]. 

MWF: Laule and colleagues examined a subgroup of PPMS patients who participated in a 2-year multi-center, randomized controlled trial, and found significantly decreased MWF from baseline to year 2 in the PPMS cohort, but not in matched healthy controls. However, the difference in change in MWF between the two groups only approached significance and no significant correlation was found between MWF and EDSS at baseline, EDSS change or T2 lesion load. All participants were on glatiramer acetate (GA), as part of a trial evaluating the efficacy of GA in PPMS. A treatment effect of GA was not detected on the change in MWF change over the study period. Despite the small sample size, this preliminary study was the first one to demonstrate a significant decrease in the SC MWF overtime [49]. Although MWF is myelin-specific, it is still not directly imaging the myelin. In this regard, UTE sequences have the potential to image the myelin proton (very short T2) in the SC WM, but this approach is still largely not investigated in the MS population [83]. 

QSM: QSM is another quantitative method that could be used for myelin-specific imaging. It uses local magnetic properties inherent in biological tissue molecular composition to quantify the relative susceptibility between tissues [81]. Wei and colleagues used a deep learning-based single-step QSM reconstruction to process the entire image [84]. This study demonstrated the potential of using QSM in imaging the SC. Although there has been recent interest in using QSM as a potential marker for assessing iron levels in the CNS, there are no studies using QSM in SC imaging in MS yet. 

### 4.3. MR Spectroscopy

Proton MRS utilizes chemical shift to analyze metabolite composition in tissue. By suppressing the overwhelming signal of water and fat acquired from a large voxel, the small signals from various metabolites can be differentiated. The spectra (whose signals are separated by parts per million (ppm) relative to the water frequency) include a measure of *n*-acetyl-aspartate (NAA), choline (cho), creatine (cr), glutamate-glutamine, and myo-inositol (M-ins), to name a few of interest to the SC MRS in MS. NAA is a marker of neuronal integrity and mitochondrial function. A large number of research studies demonstrated reduced NAA levels in the SC in patients with MS compared with controls [76,79,85,86,87,88]. The low NAA level correlates with disability measured by EDSS scores in MS, reflecting both axonal degeneration and mitochondrial dysfunction [85,86]. A recent study by Basha and colleagues in a medium cohort of MS patients found significantly lower tNAA/cr and tNAA/cho ratios as well as increased M-ins in MS patients compared to healthy controls. A significant correlation was found between tNAA/cr and EDSS score, but no correlation between tNAA/cr and CSA was observed. MRS is a valuable tool and can provide important information about MS progression, even before the actual appearance of the MS lesions and may guide treatment decisions [89]. Given this capacity, MRS is a very promising imaging modality and its use may increase in the near future. 

### 4.4. Functional MRI

Functional MRI (fMRI) explores the signal intensity changes in specific CNS regions trigged by neuronal activity. The signal intensity changes under stimuli or neuronal activity are believed to come from either the blood oxygenation level-dependent (BOLD) effect or the signal enhancement by extravascular protons (SEEP) effect. Unlike the BOLD effect, which utilizes T2* weighted signal changes associated with deoxyhemoglobin modulation in response to neuronal activities, the SEEP effect detects extracellular fluid density changes relative to neuronal activities by using PDW spin-echo acquisitions [90,91]. 

Although not used in current clinical practice, fMRI has been applied to the spinal cord in only a small number of research MS studies [92,93,94,95,96,97,98]. Interestingly, Valsasina and colleagues described increased spinal cord recruitment in both SPMS and PPMS, but increased tactile stimuli-associated cord activity in SPMS compared with PPMS patients, despite a similar SC lesion load in the two cohorts. SC fMRI abnormalities were significantly correlated with brain grey matter atrophy in the contralateral post-central gyrus only in SPMS, but not in the PPMS cohort. The tactile-associated over-recruitment in SPMS is likely caused by the altered complex excitatory and inhibitory modulation of SC interneurons [96]. 

## 5. Spinal Cord Imaging and Response to Disease Modifying Therapies

There has been increasing interest in monitoring the effect of various disease modifying therapies on tissue integrity and rate of tissue loss in the CNS. Although SC MRI data acquisition has not been considered necessary for treatment monitoring, several studies suggested that a significant proportion of MS patients could have asymptomatic SC lesions. Studies have shown that asymptomatic SC lesions are present in 27% to 53% of patients with CIS and in 25% to 32% of RRMS patients. In the last group, they are associated with older age at the disease onset, and for a substantial proportion of patients, they were the only sign of disease progression or activity. [17,99,100,101]. In this context, a small number of clinical studies, especially phase 2 studies, have incorporated SC metrics, such as atrophy, as an outcome measure, either secondary or exploratory, to monitor treatment effects. A number of technical limitations, such as the lack of reproducibility and the lack of sensitivity to small changes in the CSA, precluded widespread application. 

One of the first studies that have used CSA as a secondary outcome measure in a small cohort of 25 SPMS patients was conducted by Paolillo and colleagues more than two decades ago. The study evaluated the effect of a single pulse of the monoclonal antibody CAMPATH-1 on various MRI metrics, including SC CSA. The authors showed a significant reduction in the CSA from baseline to month 18 and a significant correlation between CSA and EDSS, but no significant treatment benefit based on CSA measurement [102]. 

Using a highly reproducible technique and studying a medium size cohort (38 patients) over four years, Lin and colleagues demonstrated the beneficial effect of subcutaneous high dose, high frequency interferon beta-1a on CSA of MS patients versus placebo and suggested that CSA may be a surrogate marker in monitoring the efficacy of disease modifying therapies [20]. In a 2-year retrospective study of 16 patients, Dupuy et al. demonstrated that IM interferon beta-1a-treated MS patients did not show any significant SC volume change compared to healthy controls, possibly reflecting a treatment effect [103]. Moreover, in a pilot 1- year study, Singhal et al. demonstrated the lack of spinal cord atrophy over time in MS patient on glatiramer acetate and the lack of a difference in CSA compared to HC, indicating a possible treatment effect. The study was limited by the small sample of 15 patients [104]. In a placebo-controlled, single center study on PPMS and transitional MS, Montalban and colleagues monitored the effect of interferon beta-1b on SC CSA on MS patients and found no difference between patients on interferon beta-1b and those on placebo over 24 months [105]. In a double-blind 2-year study in a cohort of SPMS patients, Kapoor and colleagues examined the effect of lamotrigine versus placebo on a number of MRI measures, in an attempt to study the role of sodium channel blockers on neuroprotection. The study yielded disappointing results and found no treatment benefit of lamotrigine in several primary and secondary outcomes, including the progression of SC atrophy [106]. Currently, there is an ongoing study (the FUMAPMS study) examining the effect of DMT on spinal cord CSA, as an exploratory outcome. The results have not been published yet [107].

## 6. Ultra-High Field Spinal Cord MRI in Multiple Sclerosis

After the clearance by the FDA in 2017 for limited clinical use, ultra-high field (≥7T) human MRI scanners have become clinically available. Ultra-high field imaging has higher SNR and CNR and provides more rapid high-resolution imaging which can be used to better depict fine structures of the SC than high-field (3T) and conventional (1.5T) MRI. Of note, ultra-high field imaging has increased sensitivity to gadolinium-enhancing SC lesions, given that SC tissues have longer T1 values at high fields [108]. Therefore, in order to obtain the same CNR as seen at 1.5T or 3T, a lower dose of gadolinium could be used. On the other hand, ultra-high field imaging suffers from severe radiofrequency field inhomogeneities, larger susceptibility artifacts, and higher radiofrequency energy deposition to the human body, and a lack of large field-of-view receiver coils for imaging thoracic and lumber SC. These intrinsic issues of ultra-high field systems bring technical challenges for bringing SC MRI to clinical practice. Furthermore, the lack of normative data at 7T is another obstacle to translating newly developed qualitative and quantitative metrics into clinical practice or trials. For instance, the widely used IR-based sequences for SC imaging need to be re-optimized when applied at 7T, given that some tissue properties are B_0_ dependent [109]. Several studies have demonstrated the feasibility of 7T SC imaging with generally increased SNR, CNR and lesion detectability in MS [110,111,112,113,114]. Prior comparative studies between 1.5T and 3T MRI cervical cord MRI failed to show a higher detection rate of SC lesions and Gadolinium-enhancing lesions at 3T [115,116]. Encouragingly, Dula and colleagues conducted a direct comparison of 7T and 3T cervical cord MRI and demonstrated the feasibility of 7T SC imaging with significantly improved GM-to-WM contrast and improved detection of SC lesions compared to those of 3T in patients with RRMS [112]. 

Ouellette and colleagues just published the results of a 7T imaging study demonstrating that SC lesions occur independently from brain pathology and involve both gray and WM early in the disease; surprisingly, brain pathology was more strongly related to disability than SC pathology. Their cohort of 35 patients included both RRMS and SPMS; RRMS patients developed SC lesions near the outer subpial surface, while SPMS patients developed more central lesions, showing a distinct pattern according to MS subtype [117]. Nevertheless, improved lesion conspicuity at ultra-high field may better address the clinicoradiologic paradox that warrants further investigations with more and more ultra-high field systems available.

## 7. Summary and Future Research

In summary, SC MRI plays a significant role in diagnosis and tracking disease progression in MS. Quantitative MRI techniques, particularly MTR and DTI, are promising objective measures of microstructural abnormalities in the SC in MS. Numerous studies have demonstrated that SC atrophy in MS correlates with clinical disability and disease severity in MS, and SC atrophy most likely signifies axonal degeneration that is irreversible. Ideally, we would like to have imaging measures that correlate with clinical disability prior to the development of atrophy. 

In line with the recently discovered central vein sign of MS lesions in the brain, vascular abnormalities may occur in the spinal cord but they are harder to detect [118,119,120,121]. A hybrid image method incorporating a contrast-enhanced susceptibility weighted imaging combined with T2W FLAIR has been used to highlight microvascular abnormalities in MS lesions in the brain. This approach uses an iron-based contrast agent (ferumoxytol) to enhance small vessels and improve the visibility of venous abnormalities inside MS brain lesions which are typically invisible without using this contrast agent [122]. Venous abnormalities in the SC lesions may have a similar behavior to those shown in brain lesions. Future studies employing an iron-based contrast agent to image the ‘central vein hypothesis’ in the SC are of interest to reveal microvascular involvement of the cord tissue in MS. A new potential biomarker, namely total sodium (Na) concentration, was found to be elevated in the SC of RRMS compared with healthy controls. Its measurement is feasible by MRS and may be of use in the near future [123].

The major obstacles for including spinal cord MRI scan as a routine procedure in the MS imaging are the prolonged scan time, patient discomfort, and high cost. These obstacles could be circumvented by using rapid multi-parametric quantitative MRI techniques such as synthetic MRI by Bloch equation simulation [124,125,126,127]. The Bloch simulation could be used to create a simulated PSIR image by exploiting the longitudinal magnetization without the need for phase information. This synthetic PSIR image could also improve the poor image quality when there is a thick back fat pad, which has been reported in previous studies [43,128]. A new proposed neural network algorithm can dramatically reduce the time for accurate MWF calculation and make MWF imaging more feasible and convenient [129]. A newly developed technique, based on time-efficient and robust quantitative MRI for relaxometry and PD mapping, allows whole brain coverage in 7 minutes and maybe potentially applied to SC as well [130]. These rapid quantitative MRI methods could be viable future directions in spinal cord MRI. 

## Figures and Tables

**Table 1:** Representative spinal cord quantitative MRI studies in patients with multiple sclerosis.

Article	qMRI Methods	B_0_	Study Cohorts	Main Findings
Filippi M. *Neurology* (2000) [46]	MTR	1.5T	RRMS (*n* = 52) SPMS (*n* = 33) PPMS (*n* = 11) HC (*n* = 21)	All MS vs. HC: lower MTR of the CC (*p* = 0.006), PPMS (*p* = 0.01); PPMS vs. HC: lower mean MTR (*p* = 0.01) and histogram peak height (*p* = 0.02); SPMS vs. RRMS: lower histogram peak height (*p* = 0.03).
Valsasina *p*. *NeuroImage* (2005) [47]	DTI	1.5T	RRMS (*n* = 21) SPMS (*n* = 23) HC (*n* = 17)	All MS vs. HC: decreased FA of the CC (*p* = 0.008); RRMS vs. SPMS: no difference on FA (*p* = 0.2); FA (r = −0.48, *p* = 0.001) and MD (r = 0.36, *p* = 0.02) correlate with EDSS.
Zackowski KM. *Brain* (2009) [48]	MTC	3T	RRMS (*n* = 23) SPMS (*n* = 11) PPMS (*n* = 8) HC (*n* = 18)	MS vs. HC: increased MTCSF in LC (*p* = 0.008) but not DC and GM. MTCSF of DC correlates with EDSS (r = 0.41), sway (r = 0.32), toe vibration (r = 0.58), and ankle strength (r = 0.39); MTCSF of LC correlates with EDSS (r = 0.59), ankle strength (r = −0.45) and walk velocity (r = −0.51).
Laule C. *Multiple Sclerosis Journal* (2010) [49]	MWF	1.5T	PPMS (*n* = 24) HC (*n* = 24) Longitudinal (2 years)	MS vs. HC: no difference on MWF at baseline (*p* = 0.12), but 10.5% decrement at year −2 (*p* = 0.01); No correlation for MWF vs. EDSS; Treatment vs. placebo: no difference on MWF of the cord.
Oh J. *Neurology* (2013) [50]	MTR, DTI	3T	RRMS (*n* = 69) SPMS (*n* = 36) PPMS (*n* = 19)	Low-lesion/high-EDSS vs. low-lesion/low-EDSS: decreased FA (*p* = 0.03) and MTR (*p* = 0.003); and increased MD (*p* = 0.003) and AD (*p* = 0.003); High-lesion/high-EDSS vs. high-lesion/low-EDSS: decreased FA (*p* = 0.02); increased MD (*p* = 0.02) and AD (*p* = 0.01); but no difference on MTR (*p* = 0.17).
Raz E. *American Journal of Neuroradiology* (2013) [51]	DKI, DTI	3T	RRMS (*n* = 21) HC (*n* = 16)	RRMS vs. HC: decreased FA in NAWM (*p* = 0.01); decreased MK in NAGM (*p* = 0.01); Lesions vs. NASC: decreased FA and MK (*p* < 0.001); increased MD (*p* < 0.001); High EDSS vs. low EDSS: decreased FA, MK (*p* < 0.01), increased MD (*p* < 0.01) in WC and GM but not WM; Correlation: No correlation for DTI metrics and EDSS.
By S. *NeuroImage Clinical* (2017) [52]	DTI, NODDI, DKI	3T	RRMS (*n* = 6) HC (*n* = 8)	NODDI, MS vs. HC: increased ODI in lesions (*p* < 0.001) and NAWM (*p* = 0.002); DTI, MS vs. HC: increased RD in lesions (*p* = 0.005) and NAWM (*p* = 0.01); decreased FA in lesions (*p* < 0.001) and NAWM (*p* < 0.001); DKI, MS vs. HC: decreased RK in lesions (*p* < 0.001) and NAWM (*p* = 0.016).
Collorone S. *Multiple Sclerosis Journal* (2019) [53]	NODDI	3T	RRMS (*n* = 27) HC (*n* = 18)	MS vs. HC: decreased NDI in DC, LC, GM and WC (all *p* = 0.02). EDSS correlates with NDI values of the WC (r = −0.68, *p* = 0.015) and DC (r = −0.69, *p* = 0.012).
Rasoanandrianina H. *American Journal of Neuroradiology* (2020) [54]	MTR, DTI	3T	RRMS (*n* = 13) SPMS (*n* = 6) HC (*n* = 19)	MS vs. HC: decreased IHMTR (*p* < 0.05) and MTR (*p* = <0.05) in NAWM; and decreased FA (*p* < 0.05), IHMTR (*p* < 0.001) and MTR (*p* < 0.05) in lesions; EDSS correlates with IHMTR (r = −0.73) and MTR (r = −0.81) z scores. Compared with HC, patients with MS had significantly decreased.
Oh J. *Multiple Sclerosis Journal* (2020) [55]	MTR, DTI	3T	RRMS (*n* = 45) PPMS (*n* = 30) Longitudinal (5 years)	Changes over 2 years: decreased MTR (*p* = 0.04); Changes over 5 years: decreased MTR (*p* = 0.03) and SC-CSA (*p* = 0.009); Follow-up EDSS at 2- and 5-year correlate with subject-specific changes of FA, MD, AD, MTR and SC-CSA (r = −0.5 at 2-year, r = −0.47 at 5 year).
Cortese R. *Multiple Sclerosis Journal* (2020) [56]	QSI	3T	PPMS (*n* = 23) HC (*n* = 23) Longitudinal (3 years)	Changes over 3 years in MS: increased ADC (*p* = 0.027); decreased P0 (*p* = 0.042) and CSA (*p* < 0.001); MS vs. HC: greater rate of decrease in P0 (*p* = 0.02) and CSA (*p* < 0.001); 3 years P0 changes correlates with 9-HPT (r = −0.34); 3 years FWHM correlates with 9-HPT (r = −0.24) and TWT (r = −0.46).

9-HPT = 9-Hole peg test. ADC = Apparent diffusion coefficient. AD = Axial diffusivity. RD = Radio diffusivity. B_0_ = MRI field strength. CC = Cervical cord. CSA = Cross-sectional area of SC. DC = Dorsal column. DTI = Diffusion tensor imaging. EDSS = Expanded disability status scale. FA = Fractional anisotropy. FWHM = Full width at half maximum of the displacement probability density function. HC = Healthy control. IHMTR = Inhomogeneous magnetization transfer ratio. GM = Grey matter. LC = Lateral column. MD = Mean diffusivity. MK = Mean kurtosis. MS = Multiple sclerosis. MTR = Magnetization transfer ratio. MTCSF = CSF-normalized MTC signal intensity. MWF = Myelin water fraction. NAWM = Normal-appearing white matter. NAGM = Normal-appearing grey matter. NDI = Neurite density index. NODDI = Neurite orientation dispersion and density imaging. ODI = Orientation dispersion index. P0 = zero displacement probability. PPMS = Primary Progressive MS. QSI = Q-space imaging. RD = Radial diffusivity. RK = Radial kurtosis. RRMS = Relapsing-remitting MS. SPMS = Secondary Progressive MS. TWT = 25-foot timed walk test. WC = Whole cord.
